# Crystal Structure of TNF-α-Inducing Protein from *Helicobacter Pylori* in Active Form Reveals the Intrinsic Molecular Flexibility for Unique DNA-Binding

**DOI:** 10.1371/journal.pone.0041871

**Published:** 2012-07-31

**Authors:** Mingming Gao, Defeng Li, Yonglin Hu, Ying Zhang, Quanming Zou, Da-Cheng Wang

**Affiliations:** 1 National Laboratory of Biomacromolecules, Institute of Biophysics, Chinese Academy of Sciences, Beijing, People’s Republic of China; 2 Graduate University of Chinese Academy of Sciences, Beijing, People’s Republic of China; 3 Department of Clinical Microbiology and Immunology, Third Military Medical University, Chongqing, People’s Republic of China; University of Technology Sydney, Australia

## Abstract

Tipα (TNF-α-inducing protein) from *Helicobacter pylori* is a carcinogenic effector. Studies on this protein revealed that a homodimer linked by a pair of intermolecular disulfide bridges (Cys25-Cys25 and Cys27-Cys27) was absolutely necessary for its biological functions. The activities of Tipα would be abolished when both disulfide bridges were disrupted. The crystal structures of Tipα reported to date, however, were based on inactive, monomeric mutants with their N-terminal, including residues Cys25 and Cys27, truncated. Here we report the crystal structure of *H. pylori* Tipα protein, TipαN^25^, at 2.2****Å resolution, in which Cys25 and Cys27 form a pair of inter-chain disulfide bridges linking an active dimer. The disulfide bridges exhibit structural flexibility in the present structure. A series of structure-based mutagenesis, biochemical assays and molecular dynamic simulations on DNA-Tipα interactions reveal that Tipα utilizes the dimeric interface as the DNA-binding site and that residues His60, Arg77 and Arg81 located at the interface are crucial for DNA binding. Tipα could bind to one ssDNA, two ssDNA or one dsDNA in experiments, respectively, in the native or mutant states. The unique DNA-binding activities of Tipα indicate that the intrinsic flexible nature of disulfide bridges could endow certain elasticity to the Tipα dimer for its unique bioactivities. The results shed light on the possible structural mechanism for the functional performances of Tipα.

## Introduction

*Helicobacter pylori* is a definitive carcinogen. Its infection has been associated with chronic gastritis, peptic ulcer and stomach cancer [1], [2]. Numerous proteins have been shown to participate in *H. pylori* pathogenesis. Some of these proteins, such as urease, catalase and adhesins are involved in the colonization of this bacterium in human, whereas several virulence factors are directly involved in hijacking host cells and disrupting essential cellular processes of the hijacked cells [3]–[5]. The most infamous virulence factors are the cag (cytotoxin-associated genes) pathogenicity island (cagPAI) and the vacuolating cytotoxin (VacA). *H. pylori* cagPAI encodes a complicated type IV secretion system and an effector protein CagA that is delivered into host cells by the secretion system.

During *H. pylori* infection in stomach, proinflammation cytokines such as TNF-α, IL-6 and IL-8 are induced [2], which, in turn, trigger the cytokine network as well as cellular responses [6]. These observations indicate the existence of unique *H. pylori* virulence factors other than CagA and VacA that may play pivotal roles in the course from inflammation to carcinogenesis during the infection.

Recently, a tumor necrosis factor-α-inducing protein (Tipα) was identified as a new carcinogenic factor of *H. pylori* [7]–[10]. This protein has been found to be a potent inducer of proinflammation cytokine and chemokine gene expressions [9]–[11]. It induces high expression of TNF-α through NF-κB activation and tumor-promoting activities in Bhas cells [9], [10]. TNF-α, in turn, is a well-known tumor promoter and has been identified as a master regulator of inflammation and a key player in the cytokine network between inflammation and cancer [12]. Tipα, therefore, is an important pathogenic effector of *H. pylori* that promotes host inflammation and tumor progression. The mechanism by which Tipα carries out its pathogenicity is distinct from those of well-known effectors CagA and VacA.

Tipα is secreted as homodimer by *H. pylori* independent of the type IV secretion system. The active form of Tipα can bind the gastric epithelial cells and subsequently be translocated into the cytoplasm [13]. Recently, nucleolin was reported as a membrane receptor of Tipα in gastric epithelial cells [14]. Tipα secreted from *H. pylori* acted on DNA in gastric cancer cells [15] and bound to both single-strand and double-strand forms of DNA with wide base preference *in vitro* [16], indicating that DNA binding may be involved in the molecular mechanisms of carcinogenesis.

Tipα is unique to *H. pylori* and no obvious paralogues have been found in other species so far. It is widespread in *H. pylori* strains and is encoded by gene 0596 in genome of *H. pylori* strain 26695. Tipα consists of 192 amino acids with a molecular weight of 21****KD. It has homologies of 94.3% and 95%, respectively, with proteins HP-MP1 and jph053, two counterparts from *H. pylori* strain J99. This protein is secreted from *H. pylori* as homodimer cross-linked by two inter-monomer disulfide bridges (Cys25-Cys25 and Cys27-Cys27). So far, all studies on Tipα demonstrated that the homodimer was absolutely necessary for its functional performances. Deleting both Cys25 and Cys27 would result in monomers and loss of Tipα’s bioactivities in NF-κB activation, TNF-α induction, and tumor promotion [9]–[11]. In addition, Tipα monomer has dramatically diminished DNA-binding abilities [16]. Therefore, the existence of residues Cys25 and Cys27 is essential for functional Tipα.

Because of its unique properties, the structure and structure-function relationship of Tipα have been drawing great interests. Till now, three groups have reported their crystallographic studies on this protein, but to overcome the difficulties in crystallizing the intact Tipα, they used N-terminal truncated samples that start from residue 28 (TipαN^28^) or 34 (TipαN^34^), with the critical Cys25 and Cys27 deleted [17]–[19], which, in turn, resulted in the removal of both disulfide-bridges.

Here we report the crystal structure of the N-terminal-truncated Tipα starting from the residue Cys25 (TipαN^25^) that retains both inter-molecular disulfide bridges. Our TipαN^25^ is always dimer independent of pH values or protein concentrations under non-reductive conditions. In the crystal structure, TipαN^25^ adopts an intrinsic dimeric organization as the active unit, which features two flexible disulfide bonds at the N-terminus linking two monomers of the unit to form a tweezer-like configuration. Structure-based mutagenesis and biochemical assays revealed the unique DNA-binding properties of TipαN^25^, and identified the dimer interface as the DNA-binding site and residues critical for DNA binding. It shows that Tipα binds to one ssDNA, two independent ssDNA, or one dsDNA moiety, respectively, in the native or mutant states. The observations suggest a tongs-like model in which the dimer unit adjusts the dimer interface with flexibility to certain extent so as to accommodate ssDNA and dsDNA binding in different configurations. The results shed light on the possible structural mechanisms of DNA binding for Tipα’s functions during its carcinogenesis.

## Results

### An intrinsic Dimer Linked by Disulfide Bonds

**Figure 1:**
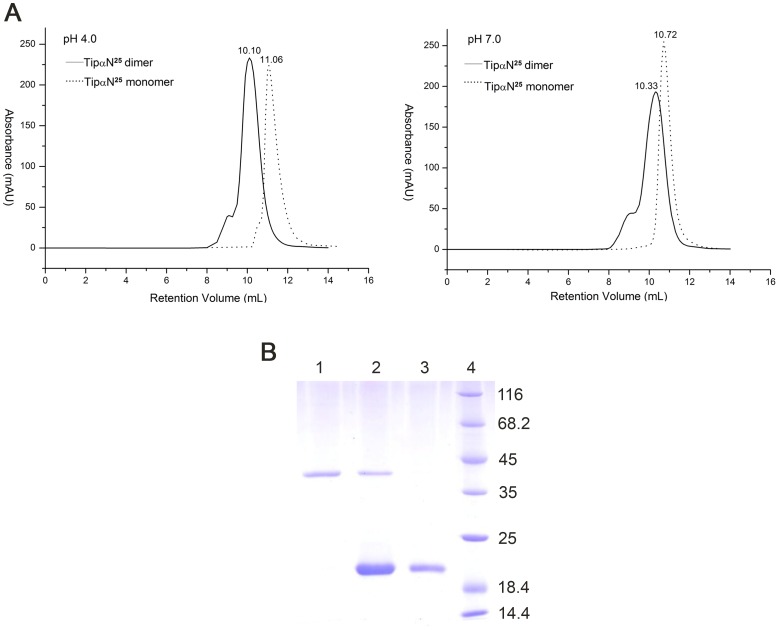
Chromatographic behaviors of TipαN^25^ at different conditions. (A) Gel filtration analysis of TipαN^25^ and TipαN^25^ monomer (TipαN^25^ treated with 50****mM DTT) at pH 4 and pH 7. (B) SDS-PAGE gel showing TipαN^25^ under different concentrations of DTT at pH 4.0. Lane 1, native TipαN^25^; Lane 2, TipαN^25^ with 10****mM DTT; Lane 3, TipαN^25^ with 50****mM DTT; Lane 4, molecular-mass-marker proteins. The sample in Lane 2 was boiled before electrophoresis, whereas those in Lane 1 and 3 were incubated at room temperature instead.

The recombinant protein TipαN^25^ (Δ1–24) includes Cys25 and Cys27 for maintaining two inter-monomer disulfide bonds. The purified TipαN^25^ at the dimeric protein concentration of 2****mg/ml displayed dimer formation under either acidic or neutral conditions as shown by analytical gel filtration experiments using Superdex 75 10/300 GL column (GE healthcare) (Fig. 1). At pH4.0 and pH7.0, TipαN^25^ eluted at a volume of 10.1****ml and 10.33****ml, respectively, both corresponding to a molecular weight of 40****KD, which was in agreement with that of a Tipα dimer. However, in the presence of 50****mM DTT, a reductant for disulfide bond, TipαN^25^ would elute at 11.06****ml at pH****4.0, corresponding to a monomeric molecular weight of 20****KD. It suggests that disulfide bonds are needed to hold the active dimeric form in the acidic gastric medium. TipαN^25^ is evidently different from previously reported TipαN^34^, which is monomer under either reducing or non-reducing conditions at pH****4 [18]. Interestingly, in pH****7.0 solutions containing 50****mM DTT, TipαN^25^ would be eluted at 10.72****ml, presenting a transitive state between dimer and monomer, which was in agreement with Tosi *et al*.’s finding that TipαN^34^ presented dimer-relevant states at pH8.5 [18]. Besides, gel filtration behaviors of TipαN^25^ at different conditions as the dimeric protein concentrations of 0.05****mg/ml and 0.25****mg/ml respectively showed the same results in Fig. S1. These experiments show that the disulfide bonds do exist in TipαN^25^ and the native TipαN^25^ always adopts a dimeric form in the absence of reducing agents, independent of protein concentrations or pH values. Studies on Tipα verified a homodimer linked by a pair of intermolecular disulfide bridges (Cys25-Cys25 and Cys27-Cys27) as the basic molecular unit that was absolutely necessary for its biological functions [9]–[11], [13], [14], [16]. Therefore, as an intrinsic dimer with disulfide bonds, TipαN^25^ used in this was an active form.

The observations indicate that disulfide bonds are essential to maintaining dimer formation of Tipα at acidic conditions; however monomeric Tipα molecules without disulfide bridges could result in dimer-relevant states in neutral or alkaline solutions. Although disulfide bonds are not necessary for keeping dimer formation under neutral or alkaline conditions, they are required for Tipα’s biological activities. Therefore, disulfide bonds are prerequisite for preserving Tipα dimer formation independent of Tipα’s charge characteristics or solution pH values and are essential to functional Tipα. The above experimental results show that the structure of TipαN^25^ reported in this study should represent an active form of Tipα.

### General Structure of TipαN^25^

#### 1) A tongs-like dimer mediated by flexible disulfide bridges

**Figure 2:**
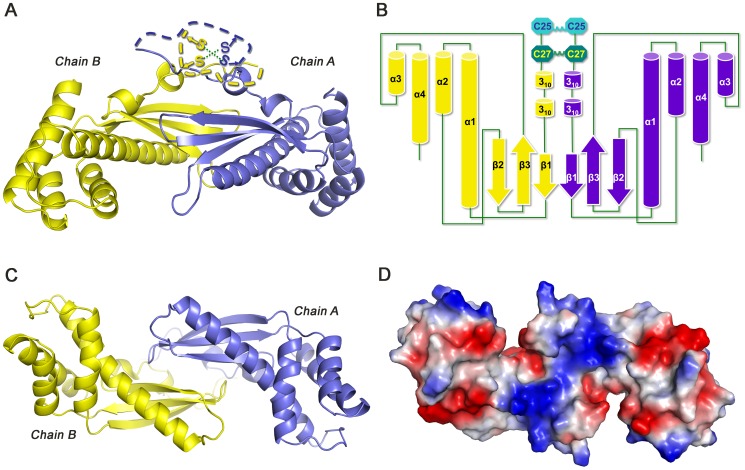
Structure of TipαN^25^ in dimeric organization with two subunits shown in purple and yellow, respectively. (A) Front view of Tipα structure. The disulfide bridges are schematically shown in the N-terminal loops. (B) Topology diagram of Tipα. (C) Top view of Tipα and its corresponding surface electrostatic potential diagram (D).

A homodimer, linked by inter-subunit disulfide bonds at the N-terminus, is present in an asymmetric unit. Two subunits of a TipαN^25^ homodimer related by a local twofold axis are arranged in an anti-parallelly way to form a unique shoulder-to-shoulder dimeric mode (Fig. 2). The N-terminal loops (25–33), where the two inter-chain disulfide bonds are located are structurally flexible and not visible in the experimental electron density map, implying their nature of intrinsic flexibility. Besides, the secondary structure prediction of Tipα showed the N-terminal 25–33 segment as a flexible loop in high confidence level of prediction, which was consistent with the disorder property of these amino acids in the structure of TipαN^25^. According to the tendency of N-terminal main chains’ extension displayed in the strucutre, we could reasonably predict that the N-terminal loops should go up around the pseudo twofold axis resulting in contacts between inter-subunit cysteines to form the disulfide bonds. Tethered by flexible disulfide bonds at the N-terminus, two subunits of TipαN^25^ are assembled in an almost anti-parallel shoulder-to-shoulder mode with the dimer interface mainly mediated through β sheets. The whole structure of TipαN^25^ homodimer resembles the shape of a pair of tongs, with the disulfide bridges as pivot and a pair of anti-parallel β sheets as jaws of the tongs to clip targets for its functional performances (Fig. 2).

#### 2) β-sheet-dominant dimeric interactions

**Figure 3:**
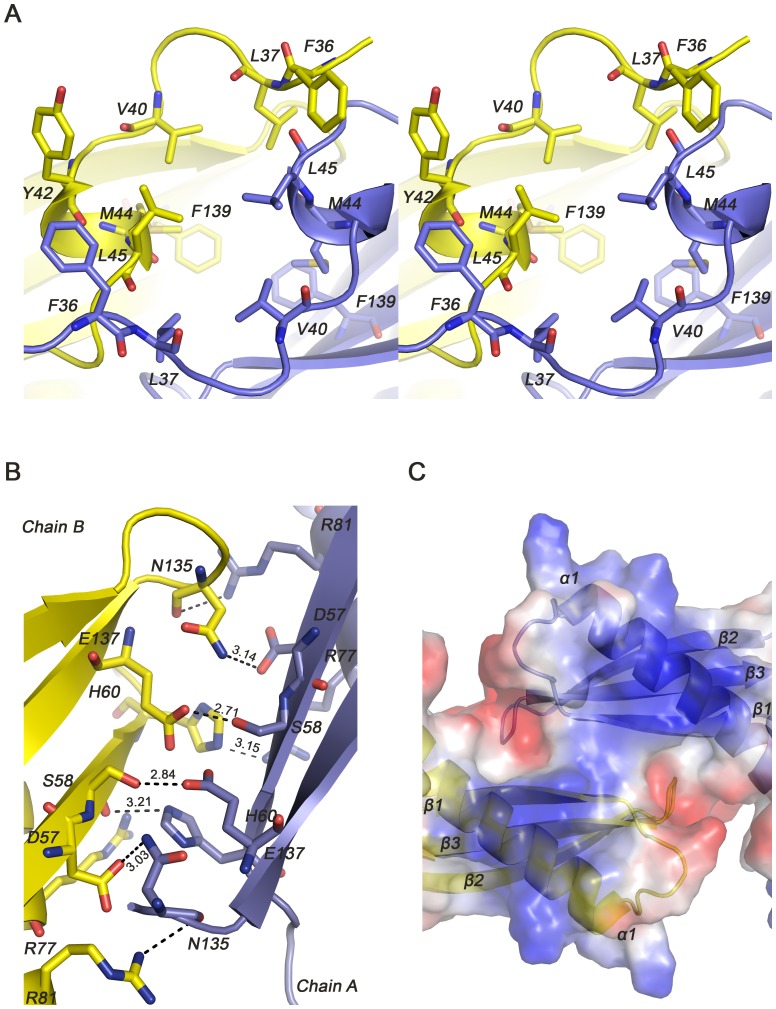
Dimeric interactions of TipαN^25^. Two subunits of Tipα (Chain A and Chain B) are shown in purple and yellow, respectively. (A) Stereo view of hydrophobic interactions of Tipα. (B) Hydrogen bond network of β-sheet-mediated interface. (C) Top view of the potential DNA-binding site at the dimer interface.

Hydrophobic interactions, hydrogen bonds, and salt bridges all contribute to dimer formation in addition to covalent disulfide bonds involving Cys25 and Cys27. Hydrophobic interactions are mainly from the N-terminal loops between residues F36, L37, V40, M44 and L45 of Chain A and residues Y42, L45, M44, V40, L37 and F36 of Chain B, as well as F139 from strand β1 of both monomers (Fig. 3A). Hydrogen-bond network and salt bridges, which strengthen inter-subunit contacts, are largely formed by β sheets. Hydrogen bonds are formed by residues D57, S58 on strand β1 with residues N135 on β2-β3 and E137 on strand β3 respectively, and R81 on helix α1 with N135 within both subunits, H60 on β1 of Chain A with S58 on β1 of Chain B, and H60 of Chain B with R77 on α1 of ChainA (Fig. 3B). The dimer interface buries a contact area of 4813.3****Å^2^, reaching 30% of the total accessible surface area, which is primarily contributed by β sheets. Therefore, the dimeric interactions of TipαN^25^ is β-sheet dominant and the dimer interface is mainly β-sheet mediated.

#### 3) Potential DNA-binding site

The surface electrostatic potential graph of TipαN^25^ demonstrated an area of concentrated positive charges at the N-terminal part of helix α1, β sheets between two subunits and part of the loops connecting stand β1 and helix α1 (Fig. 3C). In fact, Tipα monomer as an inactive form couldn’t bind receptor on host cell membrane [14] and has greatly diminished DNA-binding activities *in vitro* [16]. Thus we propose that this region, especially the β-sheet-mediated interface of Tipα dimer, provides the structural basis for DNA binding and is vital for virulent activities of Tipα.

### Structural Comparison of TipαN^25^, TipαN^28^ and TipαN^34^

**Figure 4:**
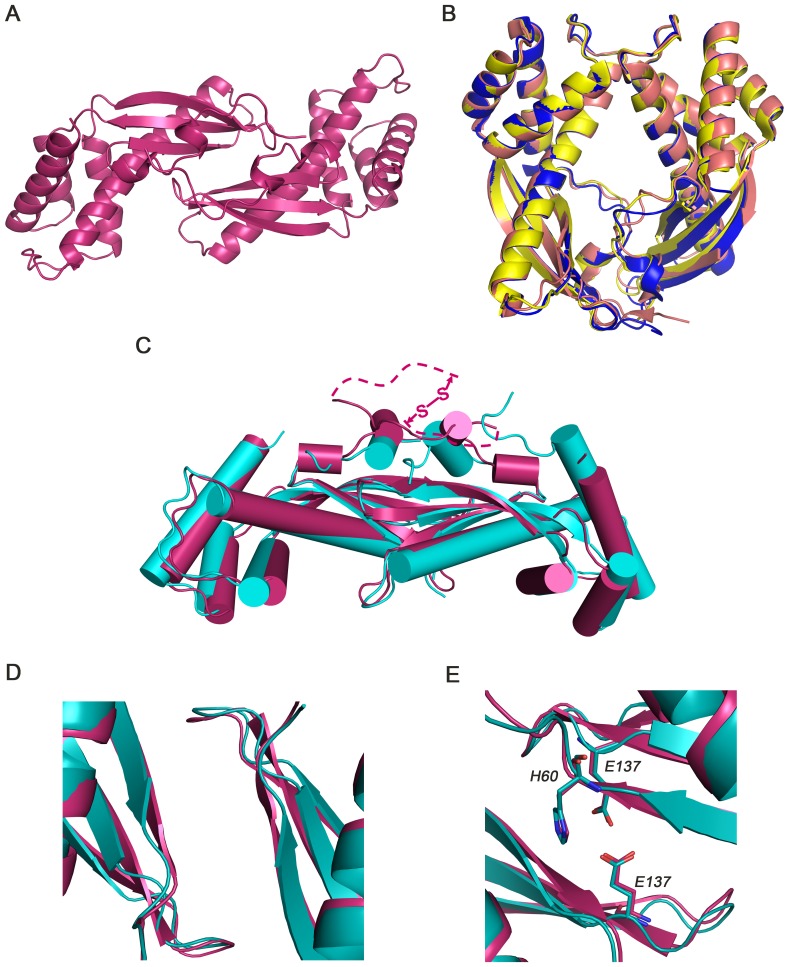
Structural comparison of TipαN^25^, TipαN^28^ and TipαN^34^. (A), (B) Dimeric organizations. TipαN^25^ takes a shoulder-to-shoulder assembly mode (A), while two TipαN^28^ and TipαN^34^-II from alkaline conditions adopt a different head-to-head dimeric mode (B). (C)- (E) Structural comparison of TipαN^25^ and TipαN^34^-I from acidic conditions. TipαN^34^-I adopts a similar dimeric organization mode with that of TipαN^25^ (C), however the dimeric β-sheets are oriented away from the dimeric interface by 10° (D) and some residue contacts involved in dimer interactions are instabilized (E) in TipαN^34^-I. TipαN^25^ (3VNC), TipαN^28^ (3GIO), TipαN^28^ (3GUQ), TipαN^34^-I (2WCQ), and TipαN^34^-II (2WCR) are coloured in red, blue, yellow, cyan and deepsalmon, respectively.

Prior to TipαN^25^, four crystal structures of N-terminal truncated mutants, *i.e.*, TipαN^28^ at pH7 [17] and at pH7.5/8.5 [19], TipαN^34^ at pH4 (TipαN^34^-I) and at pH8.5 (TipαN^34^-II) [18], have been reported, in which residues Cys25 and Cys27 were all deleted and thus inter-monomer disulfide bridges were removed. These mutants all showed dimeric organizations in an asymmetric unit and similar monomeric structures of TipαN^28^ at pH7, TipαN^28^ at pH7.5/8.5, TipαN^34^-I and II. The CA r.m.s.d.’s between TipαN^25^ and the above structures are 1.32****Å, 1.26****Å, 1.14****Å and 1.63****Å, respectively. However, the dimeric modes at alkaline conditions adopted by TipαN^34^-II and two TipαN^28^ structures, in which two monomers assemble in a head-to-head way with N-terminal loops and helices α1 and α2 to mediate the dimeric interface, are completely different from that of TipαN^25^ with inter-chain β-sheets mainly mediating the dimeric interface (Figs 4A and 4B).

Interestingly, the dimer of TipαN^34^-I at pH4 adopts a shoulder-to-shoulder assembly similar to that of TipαN^25^, but it is monomeric in solution [18]. Detailed inspection showed some subtle differences between these two dimeric structures. With the covalent linkage of disulfide bridges, two subunits of TipαN^25^ are tethered together and packed more closely than the two monomeric TipαN^34^ molecules (Fig. 4C). More importantly, the β sheets of TipαN^34^-I are oriented away from the dimeric interface by about 10° compared with those of TipαN^25^, which obviously loosen the compact dimeric interactions (Fig. 4D). Besides, residues Glu137 and His60, which are critical for the dimerization and DNA binding as identified by mutagenesis analysis, are shifted to the loops of β2-β3 and β1-β2 in TipαN^34^-I from the stands β3 and β1 of TipαN^25^, respectively, indicating structural instabilities in the dimeric interactions in TipαN^34^-I (Fig. 4E).

The observations show that in the absence of disulfide bonds, the dimeric organization modes of truncated mutants TipαN^28^ and TipαN^34^ are dependent on the acidic or alkaline pH conditions. However, in the presence of disulfide bonds, the dimeric form of TipαN^25^ exists in both crystal form and in solution, and is independent of pH values or protein concentrations. It implies that the disulfide bonds should play an important role in stabilizing the dimeric organization of Tipα. Considering that the native Tipα is secreted from *H. pylori* as homodimer with two inter-monomer disulfide bonds into the gastric medium, it is reasonable to infer that the dimeric structure of TipαN^25^ at pH4.0 should be a representative form of an active Tipα.

### DNA-binding Activities

#### 1) SsDNA binding

**Figure 5:**
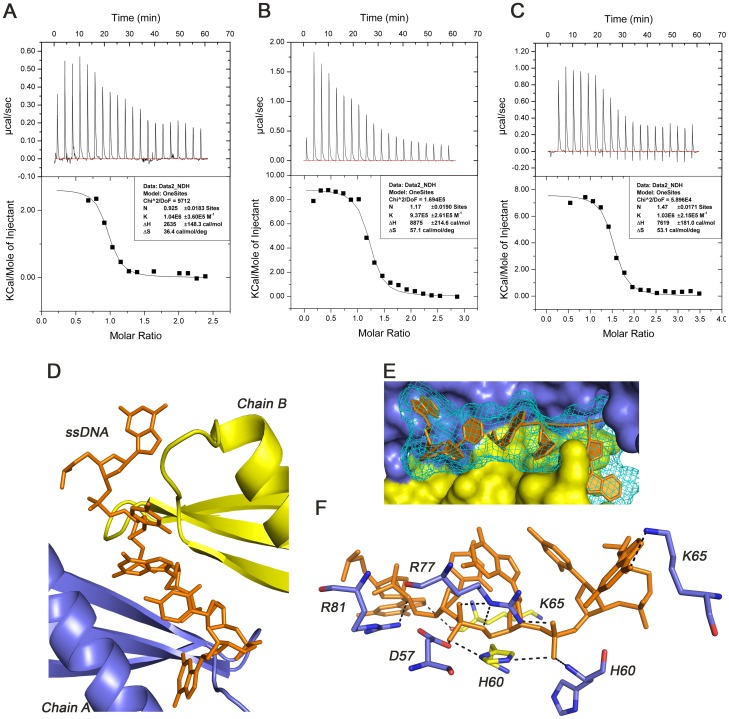
Interactions of TipαN^25^ and ssDNA. ITC experiments of Tipα with 20 nt (A), 40 nt (B), and 60 nt (C) oligomeric ssDNA. Autodock simulated model of Tipα binding an ideal ssDNA-d (GCGCG) through the dimer interface (D, E) with hydrogen bonds and salt bridges between Tipα and ssDNA (F).

To investigate the DNA-binding activities of TipαN^25^, different lengths of oligomeric ssDNA, *i.e.*, (dGdC) 10, (dGdC) 20 and (dGdC) 30, were used for binding affinity assays using ITC titration technique (Figs 5A−5C). These titrations were performed at pH5.0, which approached the acidic condition for crystal growth. The fitted parameters binding constants (K), enthalpy (ΔH), and stoichiometry (n) and the derived parameter entropy (ΔS) from these titrations were summarized in Table 1. In the experiments, Tipα samples were titrated into DNA samples and the protein concentrations were calculated based on dimeric Tipα, thus the measured stoichiometry values represented the numbers of dimeric Tipα to bind to one ssDNA.

**Table 1:** Thermodynamic parameters of Tipα with different lengths of ssDNA.

	K(M^-1^)	N	ΔH(cal/deg)	ΔS(cal/mol/deg)
(dGdC)10	1.04E6	0.925	2635	36.4
(dGdC)20	9.37E5	1.17	8875	57.1
(dGdC)30	1.03E6	1.47	7619	53.1

All the measured K values were approximately 1.0×10^6^ M^-1^, and stoichiometry (n) increased from 0.92 for (dGdC) 10 to 1.17 for (dGdC) 20, and to 1.47 for (dGdC) 30. These results indicate that one Tipα dimer could bind to one ssDNA, which implies that the dimer interface is the active binding site. Meanwhile, docking simulations performed using Autodock presented a best model, in which Tipα was binding to one ssDNA via the dimer interface, also (Figs 5D–5F). From the values of stoichiometry and the size of binding-site of Tipα structure, we infer that the Tipα dimer could cover one ssDNA with the probable longest length of 20nt.

#### 2) Essential residues for DNA binding identified by mutagenesis

**Figure 6:**
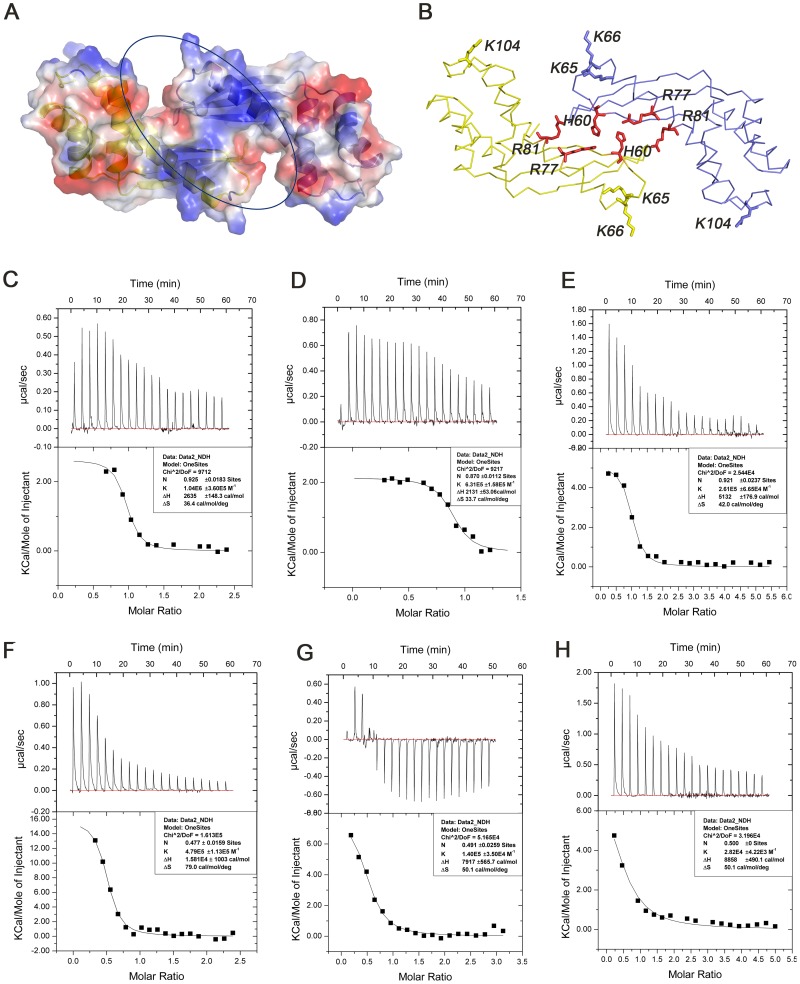
Mutagenesis analysis. (A, B) Selected amino acids for mutation on the potential DNA-binding site. ITC experiments of 20****nt oligomeric ssDNA with Tipα (C) and its mutants K104A (D), K65A/K66A (E), R77A (F), R77A/R81A (G) and H60A (H), respectively.

To further confirm the DNA-binding site of Tipα and identify the residues critical for DNA binding, structure-guided mutagenesis experiments were conducted. The Tipα-DNA docking simulation results along with the dimer interface structure suggest that the following alkaline residues His60, Arg77, and Arg81 are involved in direct contacts with the DNA phosphate backbone as well as dimeric interactions (Figs 3B and 5F). Besides, the alkaline residues Lys65, Lys66, and Lys104 are probably related to interactions with DNA. These residues were mutated into alanine to obtain five mutants, including H60A, R77A, K104A and two double mutants R77A/R81A and K65A/K66A (Figs 6A and 6B). ITC experiments were then carried out using these mutant proteins to titrate 20 nt oligomeric ssDNA (dGdC) 10 under the same condition as that of the native Tipα with (dGdC) 10 (Figs 6C-6H). Thermodynamic parameters were shown in Table 2.

**Table 2:** Thermodynamic parameters for Tipα and its mutants binding to ssDNA.

	K(M^-1^)	N	ΔH(cal/deg)	ΔS(cal/mol/deg)
Tipα	1.04E6	0.925	2635	36.4
K104A	6.31E5	0.87	2131	33.7
K65A/K66A	2.61E5	0.921	5132	42
R77A	4.79E5	0.477	15810	79
R77A/R81A	1.40E5	0.491	7917	50.1
H60A	2.82E4	0.5	8858	50.1

The binding constants K (M^-1^) listed in Table 2 showed that compared with that of wild-type Tipα (K = 1.04×10^6^), DNA-binding affinities of mutants were all evidently reduced to 1/2 for K104A (K = 6.31×10^5^), 1/3 for R77A (K = 4.79×10^5^ ), 1/5 for K65A/K66A (K = 2.61×10^5^), 1/10 for R77A/R81A (K = 1.40×10^5^) and 1/40 for H60A (K = 2.82×10^4^), respectively. These mutants, particularly H60A and R77A/R81A, in which the mutations are located at the center of the dimeric interface, have dramatically reduced DNA-biding affinities. These results demonstrate that DNA binds at the β-sheet-mediated dimeric interface of TipαN^25^ between the jaws of tongs, and residues His60, Arg77, and Arg81 are crucial for DNA binding.

#### 3) Distinct ssDNA-binding in mutants

It was very interesting to note that while the stoichiometry (n) of DNA binding was proximately 1 for wild-type Tipα and its mutants K65A/K66A and K104A, the corresponding values were about 0.5 for mutants H60A, R77A and R77A/R81A (Figs 6C-6H and Table 2). This indicates that wild-type Tipα as well as mutants K65A/K66A and K104A bind to one ssDNA per dimer, whereas mutants H60A, R77A, and R81A bind with two ssDNA, suggesting that each monomer subunit of the Tipα dimer probably binds to one ssDNA independently. In this case, the dimer for mutant H60A, R77A or R81A probably adopts an open-jaw conformation so as to accommodate two ssDNA moieties. In fact, residues His60, Arg77, and Arg81 at the dimer interface are all involved in maintaining the intensive dimeric interactions through a series of contacts, including His60-Ser58, His60-Arg77 and Arg81-Asn135 (Fig. 3B). So there is a possibility that the mutations in this case would eliminate these hydrogen bond interactions and disrupt the dimeric organization of Tipα, permitting a close-jaw to open-jaw conformational transition.

All taken into account, the experimental results demonstrate distinct ssDNA-binding properties for wild-type Tipα and its mutants H60A, R77A or R81A that are unique in binding one and two independent ssDNA, which might correspond with the rather closed and the more open dimeric conformations, respectively.

#### 4) DsDNA binding

**Figure 7:**
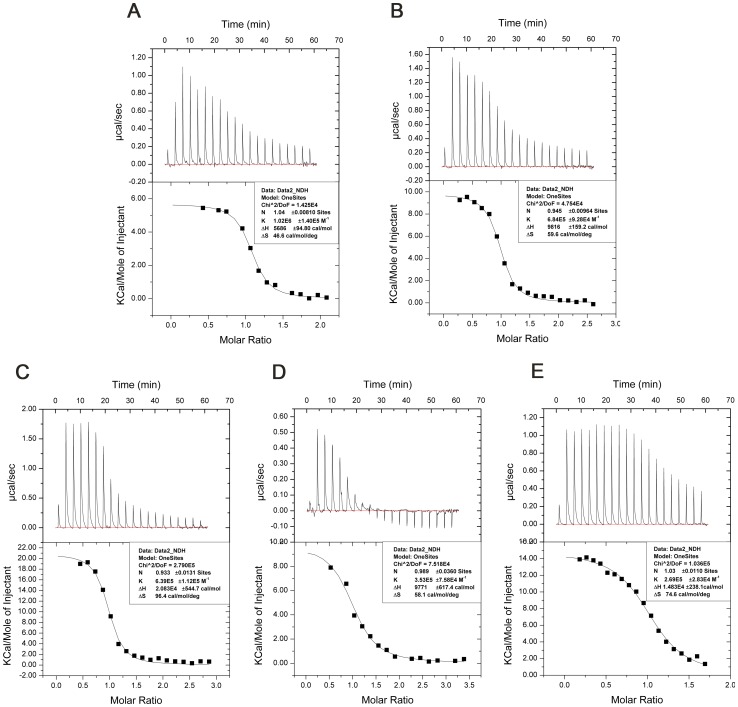
Interactions of TipαN^25^ and dsDNA. ITC experiments of 20 bp dsDNA with Tipα (A) and its mutants K65A/K66A (B), R77A (C), R77A/R81A (D) and H60A (E), respectively.

Likewise to the ssDNA-binding assay, 20 bp dsDNA were used for ITC titrations to detect dsDNA-binding characteristics of Tipα. The binding affinity of Tipα toward dsDNA (K = 1.02×10^6^ M^-1^) was measured to be almost the same as to ssDNA (K = 1.04×10^6^ M^-1^). Besides, mutations that diminished the affinities of Tipα toward ssDNA also diminished the affinities to dsDNA (Fig. 7 and Table 3). The results demonstrated that the capabilities of Tipα to bind to dsDNA and the residues critical for dsDNA binding were consistent with their ssDNA binding. Interestingly, we found that stoichiometry of dsDNA binding was proximately 1 not only for the wild-type Tipα and mutant K65A/K66A, but also for mutants H60A, R77A and R77A/R81A, illustrating that these dimeric proteins all bound to one dsDNA. In summary, the wild-type Tipα could bind to either one dsDNA or one ssDNA; while mutants H60A, R77A, and R81A could accommodate the binding of either one dsDNA or two ssDNA. Considering that the wild-type structure of Tipα we obtained was suitable for the binding of one ssDNA, but not for the binding of dsDNA, we therefore propose that dsDNA could induce a specific conformational change at the dimer interface from that of a compact to a more open state when binding occurs.

**Table 3:** Thermodynamic parameters for Tipα and its mutants binding to dsDNA.

	K (M^-1^)	N	ΔH (cal/deg)	ΔS (cal/mol/deg)
Tipα	1.02E6	1.04	5686	46.6
K65A/K66A	6.84E5	0.945	9816	59.6
R77A	6.39E5	0.933	20830	96.4
R77A/R81A	3.53E5	0.989	9771	58.1
H60A	2.69E5	1.03	14830	74.6

SPR experiments were conducted at pH7.0 to measure interactions between DNA and native Tipα as well as the key mutant H60A, because the heat was too small to calculate the affinities of Tipα and the mutant proteins toward DNA at pH7.0 in ITC assay. The SPR assay results showed that the KD value of Tipα protein to 20 nt oligomeric ssDNA (dGdC) 10 was 1.12 µM, while that of H60A was 60.3 µM, indicating that the binding affinity of Tipα to oligomeric (dGdC) 10 ssDNA at pH 7.0 was nearly the same as that at pH 5.0 in ITC assay but 54 times stronger than that of H60A at pH 7.0 as shown in Table 4 and Fig. S2. The SPR experiment results at pH7.0 were in agreement with their corresponding ITC experiments at pH5.0. Therefore, taken all together, DNA binding activities of Tipα are pH independent and our structure-based ITC assay results are representative.

**Table 4:** SsDNA binding activities of Tipα and H60A in SPR assay.

	Tipα	H60A	ratio(Tipα/H60A)
KD (µM)	1.12	60.3	54
ka (1/Msec)	598	2.9	206
kd (1/sec)	6.73E-04	1.75E-04	3.84

**Figure 8:**
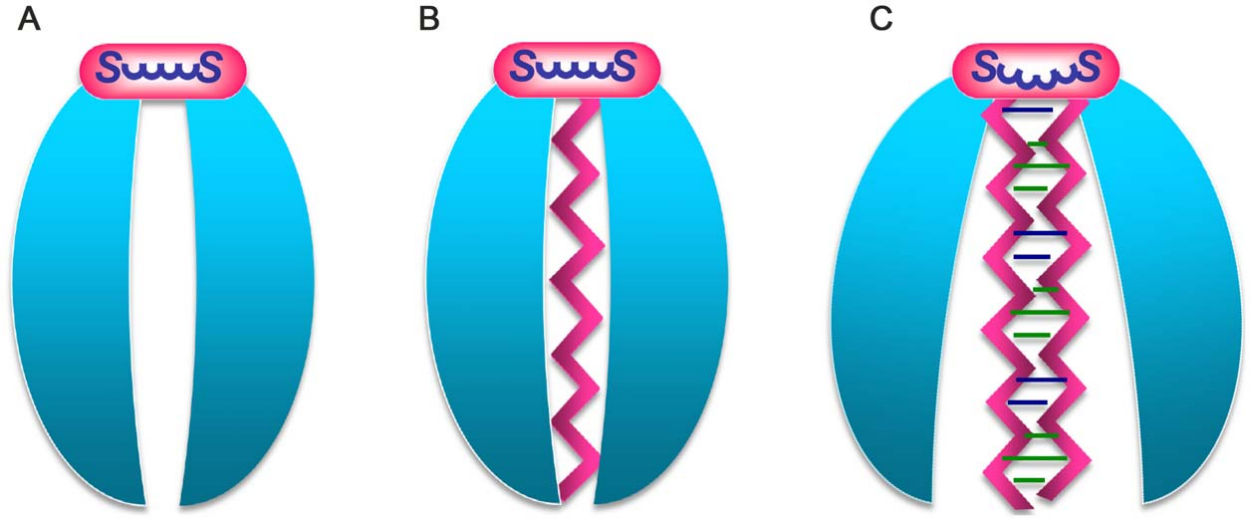
Schematic view showing conformational changes of Tipα in DNA binding mediated by flexible disulfide bridges. (A) Native Tipα dimer model. (B) SsDNA binding with the wild-type Tipα. (C) Conformational change of Tipα induced by dsDNA binding.

The native Tipα displays unique DNA-binding activities for both ssDNA and dsDNA, represented by the compact wild-type structure of TipαN^25^ and a more open dimeric structure, respectively (Fig. 8). The unique DNA-binding activities reveal the intrinsic molecular flexibility of Tipα.

## Discussion

### Dimerization Propensity

It is well known that the dimer with inter-subunit disulfide-bridge linkage is the active unit of functional Tipα [9]–[11], [13], [14], [16]. While without disulfide bonds monomeric Tipα could also form dimeric-relevant states in neutral or alkaline solutions as shown in this report and in Ref. 18. These observations indicate that at neutral or alkaline conditions, disulfide-bridge-disrupted or Cys-deleted monomeric Tipα possesses the dimerization propensity. Besides, distinct dimeric modes are displayed in the crystal structures of monomeric TipαN^28^ and TipαN^34^ depending on the acidic or alkaline pH. Thus in general disulfide bridges are not the only factor for dimerization of Tipα, if we don’t take the requirements of the functional performances of active Tipα into account. On the other hands, the dimerization behavior of TipαN^25^ is independent of pH values or protein concentrations in solution or during crystallization, which shows an intrinsic dimerization property endowed by the disulfide bridges. The 3D structure of this TipαN^25^ with disulfide bridges at pH4.0 adopts the “shoulder-to-shoulder” dimerization mode that is the same as that of TipαN^34^ structure at acidic pH. Considering that there is no report until now to show that the dimeric form of Tipα with the disulfide bonds removed could retain its bioactivities, the structure of our TipαN^25^ with disulfide bridges represents an active form of Tipα and the disulfide bridges involving Cys25 and Cys27 play a pivotal role in stabilizing the active homodimer organization for its functional performances.

### Intrinsic Elasticity of Tipα Mediated by Flexible Disulfide Bridges

Disulfide bridges are required and absolutely necessary for Tipα’s biological activities [9]–[11], [13], [14], [16]. In the structure of TipαN^25^ as reported in this study, these disulfide bridges are disordered and can not be built into the structural model, although their existence is proved by the solid data. It is quite interesting to know what roles this ‘recluse’ plays in the structural and functional properties of Tipα. The detailed analyses on the TipαN^25^ structure in correlation with its unique DNA-binding properties provide us with clues to this ‘puzzle’: the Tipα dimer is endowed with the intrinsic elasticity by the flexible disulfide bridges and N-terminal loops for its specific biological functions.

In the present study, the experiments revealed the unique DNA-binding properties for TipαN^25^. The wild-type Tipα could bind to one ssDNA or one dsDNA; however mutants H60A, R77A, and R81A could accommodate two ssDNA or one dsDNA. Considering that these target DNAs have different configurations, it should require variable conformational states of Tipα in response to corresponding DNA substrates. For the wild-type Tipα, the structure of TipαN^25^ reported in this study may be a protomeric state for one ssDNA binding, and the dsDNA binding would in some way induce a close-jaw to an open-jaw conformational transition. As key residues of His60, Arg77 and Arg81 for maintaining the compact dimeric interface of Tipα, mutations to alanine for them would disrupt these intensive dimeric interactions, thus these mutants probably accommodate two ssDNA moieties or the dsDNA moiety through a relatively loose dimeric conformation. In all of these changes Tipα should keep the active dimeric form maintained by disulfide bridges. The comprehensive observations indicate that the native Tipα should possess intrinsic elasticity mediated by the flexible disulfide bridges as schematically shown in Fig. 8, which may provide the structural basis for conformational changes in response to the requirements of different functional performances of Tipα. To verify these proposals, further experiments, such as the 3D structures of Tipα-DNA complexes are certainly needed.

### Functional Implication of the Elastic Tipα Dimer

In this study the structure of TipαN^25^ together with its specific DNA-binding properties revealed the unique structural features of an active form in dimeric form with disulfide bridges. In the native TipαN^25^ structure, two monomers are tethered by the flexible disulfide bridges as pivot to form a tong-like configuration, and the β-sheet-dominant dimer interface resembles the jaws of the tongs to ‘clip’ corresponding targets. In this way, flexible disulfide bonds could provide certain elasticity to enable the dimer interface to adjust itself for different targets, such ssDNA, dsDNA or membrane receptor nucleolin of gastric epithelial cells. Thus the intrinsic dimer-flexibility and elasticity of Tipα provides the structural basis for its biological activities.

Tipα was identified as a new carcinogenic factor for induction of high expression of TNF-α during *H. pylori* infection. However, the main molecular events and the specific target and effector proteins involved in these biological processes have not been identified. The observations reported in this study reveal new clues in understanding its carcinogenic mechanisms. Probably after entering the nucleus, the active dimeric Tipα might recognize and bind to DNA targets to initiate transcription of TNF-α, which could then active NF-κB thus positively regulating TNF-α and chemokine gene expressions. Tipα might directly or indirectly active transcription of TNF-α, other pro-inflammation cytokines and chemokines to mediate its functional process related with carcinogenesis.

## Materials and Methods

### Preparation, Expression and Purification of Recombinant TipαN^25^

The DNA fragment of TipαN^25^ was amplified from *H. pylori* 26695 genomic DNA and then was cloned into pET22b (+) vector (Novagen) placed between *Nde*I and *Xho*I restriction sites. A six-histidine (LE-6×H) tag was engineered into the C-terminus of the protein.

Recombinant TipαN^25^ protein was expressed in *E. coli* BL21 (DE3) at 16°C, 100 µΜ isopropyl-β-D-galactopyranoside for 20****h after culture growth for 2****h at 37°C. TipαN^25^ with SeMet incorporated (SeMet TipαN^25^) was over-expressed in *E. coli* B834 cells using M9 culture medium supplemented with SeMet before induction and induced in the same conditions as that of native TipαN^25^ protein.

The protein was loaded on Ni-NTA column (GE Healthcare) previously equilibrated with lysis buffer (50****mM****NaH_2_PO_4_ pH****8.0, 300****mM****NaCl, and 10****mM imidizole). The column was then washed with wash buffer (50****mM****NaH_2_PO_4_ pH8.0, 300****mM****NaCl, and 20****mM imidizole), and protein samples were subsequently eluted with elution buffer (50****mM****NaH_2_PO_4_ pH8.0, 300****mM****NaCl, and 250****mM imidizole). The protein was further purified with cation-exchange chromatography using a Hitrap SP column (GE Healthcare) to remove the non-specifically bound nucleic acids, and the protein was eluted with a linear gradient generated by Buffer A (25****mM****Na/K at pH****6.2) and Buffer B (25****mM****Na/K pH****6.2, 2****M****NaCl). The protein samples were then applied onto a Superdex 75 column (GE healthcare) for size-exclusion chromatography and eluted with the crystallization buffer (25****mM Bis-tris pH****6.8, 150****mM****NaCl). The proteins of SeMet TipαN^25^ and TipαN^25^ mutants were purified using the same protocol as native TipαN^25^.

### Crystallization

The hanging-drop vapor diffusion method was used for the crystallization of native TipαN^25^ protein. Crystals were obtained by mixing equal volumes of protein (20****mg/mL) and reservoir solution containing 8% Tacsimate pH3.5, 2% 1,2-propanediol (v/v), 5% MPD (v/v), 16% of PEG3350 (w/v), and 5%–10% glycerol (v/v). Crystals of SeMet TipαN^25^ (8****mg/mL) grew in 8% Tacsimate at pH4.0, 2% 1, 2-propanediol (v/v), 5% DMSO (v/v), and 16% of PEG3350 (w/v) using micro-batch method.

### Structure Determination

All diffraction data sets were collected at Beamlines NW12 and 17A of Photon Factory, KEK, Japan. The native and Se-Met crystals diffracted to 2.2****Å and 2.6****Å respectively, and both belonged to the space group C2, with unit cell parameters of a = 138.7****Å, b = 46.94****Å, c = 99.1****Å, α = γ = 90°, β = 127.8° for the SeMet (peak) data. The diffraction images were processed by Mosflm [20] and Scala [21]. The asymmetric unit contains a homodimer molecule of Tipα with a solvent content of 58.6%. Multiple anomalous dispersion (MAD) method was used to calculate the experimental phases [22]. The selenium sites were determined using program ShelX and then phases were obtained and improved by SOLVE and RESOLVE [23]. The initial model was built using Arp-wArp [24], and subsequently completed manually using COOT [25]. The structural refinement was performed using the program CNS [26]. The final R_work_ and R_free_ [27] of the model were 23.1% and 26.4%, respectively. The quality of the model was evaluated by PROCHECK [28], and it was found that 93.8% amino acid residues were in the most favoured regions. The N-terminal 9 amino acid residues (C25-R33) and the C-terminal 10 amino acids (Y183-M192) were not observed in the experimental electron density map. The data collection and refinement statistics were summarized in Table 5. All structure pictures were drawn in PyMol (http://pymol.org/).

**Table 5:** Data collection and refinement statistics.

	Se			Native
	Peak	Infl	RemoteH	
Data collection				
Space group	C2			C2
Cell dimensions				
a, b, c (Å)	138.7, 46.93, 99.1	138.71, 46.91, 99.06	139.21, 47.08, 99.07	127.01, 47.47, 96.5
α, β, γ	90, 127.78, 90	90, 127.76, 90	90, 127.68, 90	90, 127.5, 90
Wavelength (Å)	0.97898	0.97917	0.96395	0.96409
Resolution range	49.15–2.6	49.15–2.65	49.15–2.7	33.89–2.2
*R*_merge_ (%)^a^	10.2 (37.4)	10.9 (40.8)	11.7 (40.7)	6.9 (38.8)
*I*/σI*	17.7 (4.7)	17.8 (4.0)	11.2 (2.8)	13.1 (3.9)
Completeness (%)^a^	99.9 (99.9)	99.8 (100)	99.8 (100)	97 (82.8)
Redundancy^a^	7.2 (7.3)	7.1 (7.4)	3.6 (3.6)	4.6 (3.3)
Refinement				
No. reflections	15054			
*R*_work_/*R*_free_ (%)	23.1/26.4			
Averaged B-factors (Å)	48.7			
No. of Assymtry Unit	1			
No. of protein atoms	2460			
No. of water	59			
R. m. s. divisions				
Bond lengths (Å)	0.009			
Bond Angels (°)	1.3			
Ramachadran plot				
Most favored region (%)	93.8			
Additional allowed region (%)	5.4			
Disallowed region (%)	0.7			

a The values in parentheses are statistics from the highest resolution shell.

### Isothermal Titration Calorimetry

ITC titrations [29] were performed at 25.0°C on an ITC200 (GE Healthcare). The buffer for both protein samples and oligomeric ssDNA (dGdC) 10, (dGdC) 20, and (dGdC) 30, and oligomeric dsDNA (GCCTTGCCGCCGCCCTTGCC) contained 50****mM Citric acid and Na Citrate pH5.0, 150****mM****NaCl. The protein concentrations were for dimeric TipαN^25^ or its mutants. During titration, 1–2****µL aliquots of protein were injected (20 injections) regularly from a rotating syringe into 200****µL of DNA solution in an isothermal calorimeter cell. Control experiments were conducted under the same conditions using buffer solution instead of DNA (Fig. S3). The dilution heat of protein from the control experiment was then subtracted to obtain the isotherm. Binding isotherms were fitted with Origine 7.0 (http://www.originlab.com/) using one set of sites model.

### Surface Plasmon Resonance

SPR [30] experiments were carried out at 25°C using a BIAcore T100 (BIAcore AB, Sweden). The HPLC-purified 5′-biotine-labeled DNA of 20****nt (dGdC) 10 was immobilized on a streptavidin (SA)-coated sensor chip. Flow cell 1 was left blank, while flow cell 2 was immobilized with (dGdC) 10. The buffer for both protein samples and experiment flow-through running was 50****mM Hepes at pH7.0, 150****mM****NaCl and 0.005% Tween20. Protein samples were injected at different concentrations at a flow rate of 30****µl/min for 1 min.

### Molecular Docking

Autodock is an automated procedure for predicting optical conformations and orientations for the ligand, protein or DNA with the target proteins at the binding site [31], [32]. Tipα-DNA docking simulations were performed using Autodock version 4.2 (http://autodock.scripps.edu/) with the Lamarckian Genetic algorithm (LGA) method. Polar hydrogen atoms were added to the target protein Tipα and its nonpolar hydrogens were merged. Ideal oligomeric ssDNA - (dGCGCG) was treated as flexible ligand and only torsions of freedom were explored, keeping both bond angles and lengths constant. The grid box was centered on Tipα with a dimension of 100×100×100 points. Each docking simulation was repeated 20 times using different random generator seeds. The interactions of complex Tipα-DNA conformations were analyzed using Pymol.

### Protein Data Bank Accession Number

Coordinates and structure factors for the structure of TipαN^25^ have been deposited to Protein Data Bank with the accession code 3VNC.

## Supporting Information

Figure S1 **Chromatographic behaviors of TipαN^25^ as the dimeric protein concentrations of 0.05 mg/ml and 0.25 mg/ml respectively at different conditions.** Different protein concentrations of TipαN^25^ at 0.05****mg/ml and 0.25****mg/ml were respectively used, and meanwhile TipαN^25^ dimer sample was reduced in the presence of 50****mM DTT to obtain TipαN^25^ monomer. Gel filtration analysis of TipαN^25^ and corresponding TipαN^25^ monomer at pH4 as the dimeric protein concentration of 0.05****mg/ml (A) and 0.25****mg/ml (B), respectively; Gel filtration analysis of TipαN^25^ and corresponding TipαN^25^ monomer at pH7 as the dimeric protein concentration of 0.05****mg/ml (C) and 0.25****mg/ml (D), respectively. (E) Typical chromatogram of various molecular-weight proteins in Superdex 75 10/300 GL column (http://www.gelifesciences.com/).
(TIF)

Figure S2 **DNA-binding analysis of Tipα and mutant H60A with 20 nt oligomeric ssDNA in SPR assay.**
(TIF)

Figure S3 **Control experiments with proteins titrating solution buffer before corresponding protein-DNA interactions in ITC assay.** Controls of Tipα with 20 nt, 40 nt, and 60 nt oligomeric ssDNA shown in (A), (B), and (C), respectively. Controls of mutants K104A (D), K65A/K66A (E), R77A (F), R77A/R81A (G) and H60A (H) with 20 nt oligomeric ssDNA, respectively. Controls of proteins Tipα (I), K65A/K66A (J), R77A (K), R77A/R81A (L) and H60A (M) with 20bp oligomeric dsDNA, respectively.
(TIF)
