# Supplementary figures and images for: Crystal Structure of TNF-α-Inducing Protein from Helicobacter Pylori in Active Form Reveals the Intrinsic Molecular Flexibility for Unique DNA-Binding
Source: PLoS One. 2012 Jul 31;7(7):e41871. doi: 10.1371/journal.pone.0041871 (PMC3409205; doi:10.1371/journal.pone.0041871)

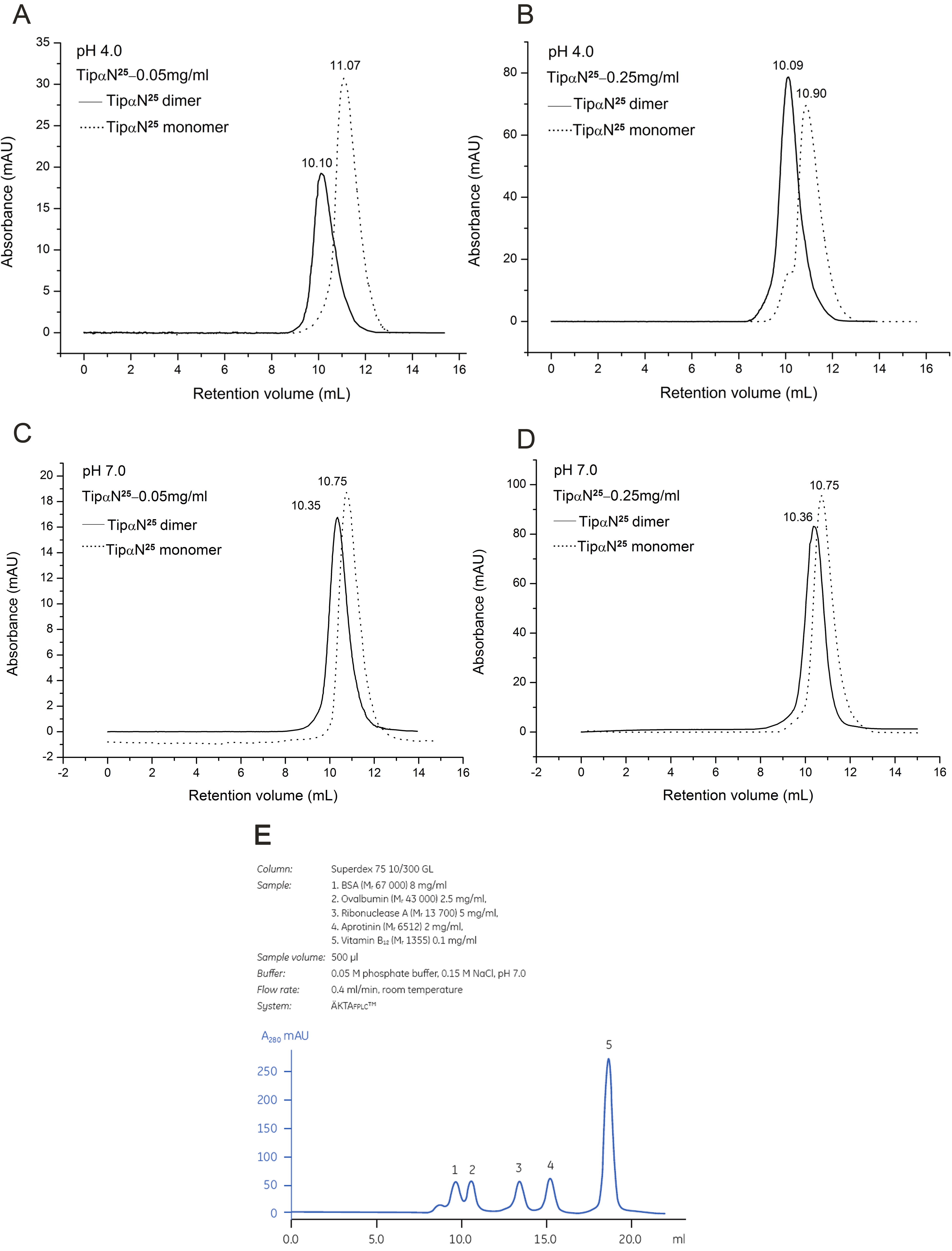

Supplement: Figure S1 — Chromatographic behaviors of TipαN25 as the dimeric protein concentrations of 0.05 mg/ml and 0.25 mg/ml respectively at different conditions. Different protein concentrations of TipαN25 at 0.05mg/ml and 0.25mg/ml were respectively used, and meanwhile TipαN25 dimer sample was reduced in the presence of 50mM DTT to obtain TipαN25 monomer. Gel filtration analysis of TipαN25 and corresponding TipαN25 monomer at pH4 as the dimeric protein concentration of 0.05mg/ml (A) and 0.25mg/ml (B), respectively; Gel filtration analysis of TipαN25 and corresponding TipαN25 monomer at pH7 as the dimeric protein concentration of 0.05mg/ml (C) and 0.25mg/ml (D), respectively. (E) Typical chromatogram of various molecular-weight proteins in Superdex 75 10/300 GL column (http://www.gelifesciences.com/). (TIF) [file pone.0041871.s001.tif]

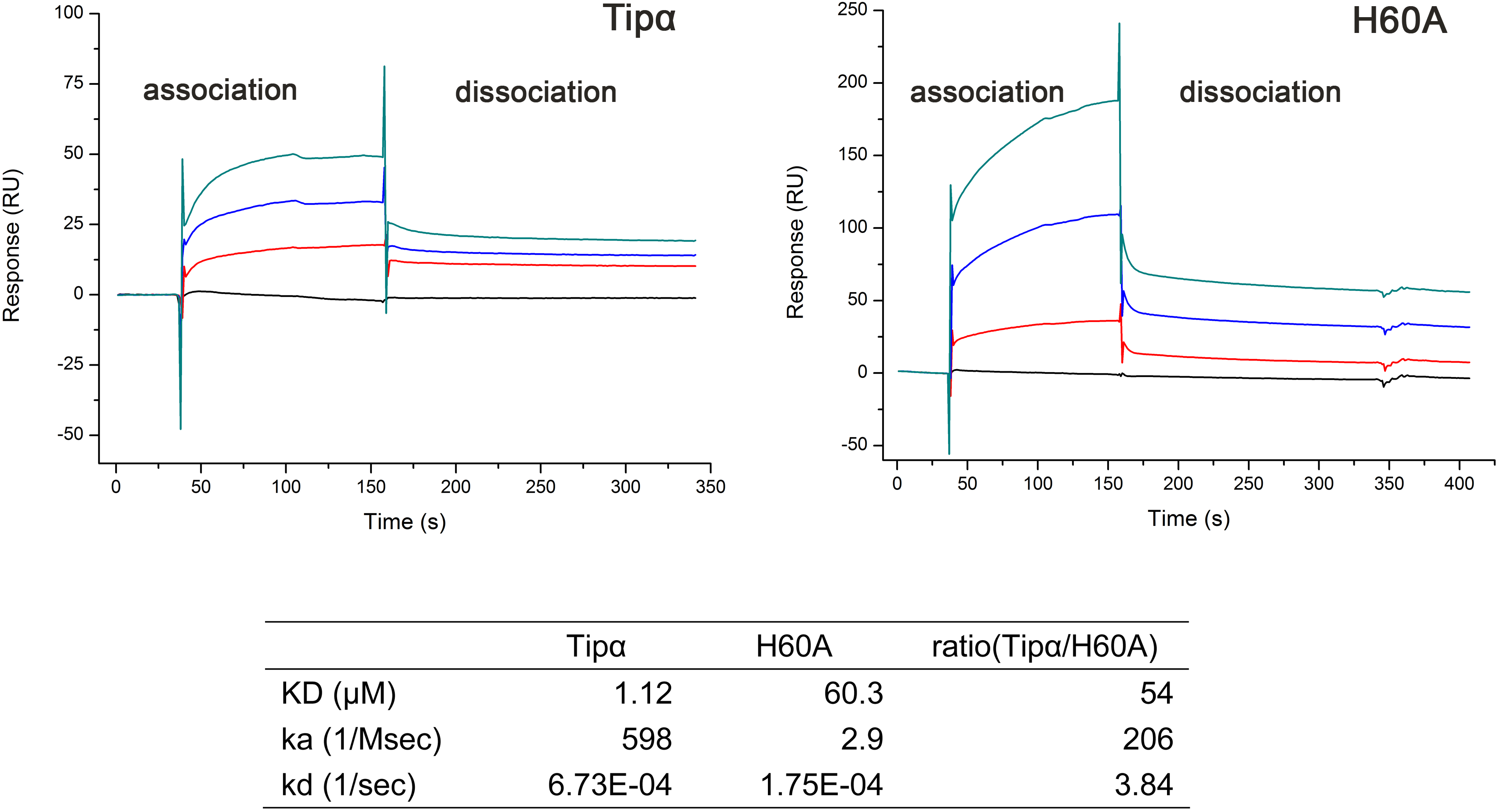

Supplement: Figure S2 — DNA-binding analysis of Tipα and mutant H60A with 20 nt oligomeric ssDNA in SPR assay. (TIF) [file pone.0041871.s002.tif]

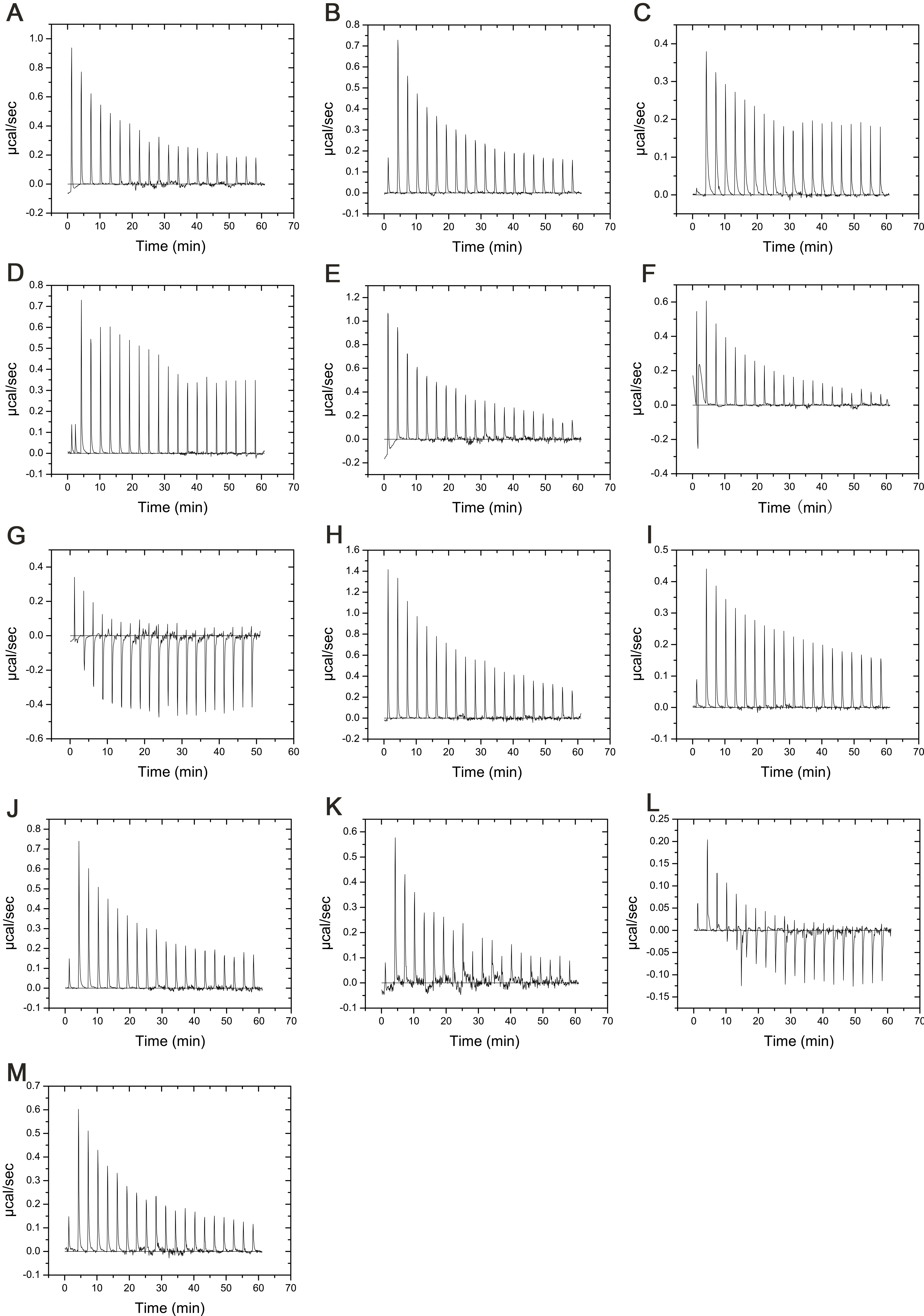

Supplement: Figure S3 — Control experiments with proteins titrating solution buffer before corresponding protein-DNA interactions in ITC assay. Controls of Tipα with 20 nt, 40 nt, and 60 nt oligomeric ssDNA shown in (A), (B), and (C), respectively. Controls of mutants K104A (D), K65A/K66A (E), R77A (F), R77A/R81A (G) and H60A (H) with 20 nt oligomeric ssDNA, respectively. Controls of proteins Tipα (I), K65A/K66A (J), R77A (K), R77A/R81A (L) and H60A (M) with 20bp oligomeric dsDNA, respectively. (TIF) [file pone.0041871.s003.tif]
